# 906. Retrospective Study of Treatment and Outcomes in Pediatric Hand Burns

**DOI:** 10.1093/jbcr/irag033.410

**Published:** 2026-04-06

**Authors:** Joyce J Zhu, Nicolette Bullard, Aiden Jaskolka-Brown, Adriana Baez, Jesseca Antoine, Chloe E Chapin-Grabowski, Sai Ram Velamuri

**Affiliations:** University of South Florida Health Morsani College of Medicine, Tampa, FL; University of South Florida Health Morsani College of Medicine, Tampa, FL; University of South Florida Health Morsani College of Medicine, Tampa, FL; University of South Florida Health Morsani College of Medicine, Tampa, FL; University of South Florida Health Morsani College of Medicine, Tampa, FL; Tampa General Hospital, Tampa, FL; University of South Florida Health Morsani College of Medicine, Tampa, FL

## Abstract

**Introduction:**

Examine various treatment patterns and long-term patient outcomes of pediatric hand burns, with a focus on the prevalence of scar contracture and need for subsequent therapies.

**Methods:**

A retrospective cohort study was completed on patients under the age of 18 that presented with hand burns between January 2021 and December 2024. Variables collected include patient demographics, surgical interventions, acute and long-term treatments received, and occurrence of complications including seroma, hematoma, infection, graft loss, dehiscence, and contracture. Statistical analyses using t-tests, Fisher’s exact, and multivariable logistic regressions were conducted in R.

**Results:**

A total of 475 pediatric burn patients were assessed, 158 cases included hand involvement (n = 196 hand injuries), with 38 bilateral hand injuries and 106 requiring hospital admission. During hospital admission, 100 patients participated in occupational therapy, 36 patients required splint usage, and 39 patients underwent surgery (skin grafting or debridement). A total of 11 complications (seroma, hematoma, graft loss, dehiscence) and 14 hand contractures were observed. Follow-up treatment included steroid injections (n = 7), laser therapy (n = 11), and contracture release surgery (n = 8). Burn depth was significantly associated with contracture formation (*p*<.001), steroid injection use (*p*=.001), laser therapy use (*p*=.019), and contracture release surgery (*p*=.003). After propensity score-matching, splint use was associated with higher odds of contracture (adjusted OR 19.8, *p*<.001). Contracture rates decreased over time, with Firth logistic regression indicating lower odds of contracture in 2024 compared with earlier years (adjusted OR 0.21, *p*=.020). Age and sex were not significantly associated with contracture.

**Conclusions:**

Hands are a common burn site in pediatric populations and proper rehabilitative interventions are essential to preserve functionality. Burn depth is a key predictor of contracture and need for additional subsequent interventions. While splinting correlated with higher contracture rates, this may reflect confounding by indication.

**Applicability of Research to Practice:**

No standardized rehabilitation protocol exists for the management of pediatric hand burns. This work provides insights into our management strategies. Moreover, the efficacy of splinting in preventing burn contractures in pediatric patients remains controversial. Our findings highlight the need for further studies to develop effective practice guidelines to improve long-term outcomes for the pediatric burn population.

**Funding for the study:**

N/A.

